# Comparative Accuracies of the N-Localizer and Sturm-Pastyr Localizer in the Presence of Image Noise

**DOI:** 10.7759/cureus.9137

**Published:** 2020-07-11

**Authors:** Armando L Alaminos-Bouza, Russell A Brown

**Affiliations:** 1 Medical Physics, MEVIS Informática Médica Ltda., São Paulo, BRA; 2 Principal Engineer, Retired, Palo Alto, USA

**Keywords:** stereotactic surgery, stereotactic radiosurgery, computed tomography, magnetic resonance imaging, n-localizer, sturm-pastyr localizer, monte carlo, image-guided surgery, image-guided radiosurgery, deep brain stimulation

## Abstract

The N-localizer and the Sturm-Pastyr localizer are two technologies that facilitate image-guided stereotactic surgery. Both localizers enable the geometric transformation of tomographic image data from the two-dimensional coordinate system of a medical image into the three-dimensional coordinate system of the stereotactic frame. Monte Carlo simulations reveal that the Sturm-Pastyr localizer is less accurate than the N-localizer in the presence of image noise.

## Introduction

The N-localizer was introduced in 1979 [1], and the Sturm-Pastyr localizer was introduced in 1983 [2]. Both localizers enable the geometric transformation of tomographic image data from the two-dimensional $\left ( u, v \right )$ coordinate system of a medical image into the three-dimensional $\left ( x, y, z \right )$ coordinate system of the stereotactic frame. Geometric transformation requires calculations that differ substantially between the two localizers in ways that impact the accuracy of the calculations when the effects of image noise are considered.

## Technical report

Geometric transformation requires the calculation of $\left ( x, y, z \right )$ coordinates in the three-dimensional coordinate system of the stereotactic frame. The following presentation discusses the calculation of only the $z$-coordinate because the calculation of the $\left( x, y \right)$ coordinates is trivial due to features of the N-localizer and Sturm-Pastyr localizer. Specifically, the N-localizer includes two vertical rods that have fixed values of $x$ and $y$, and the Sturm-Pastyr localizer includes one vertical rod that has fixed values of $x$ and $y$.

**Figure 1:**
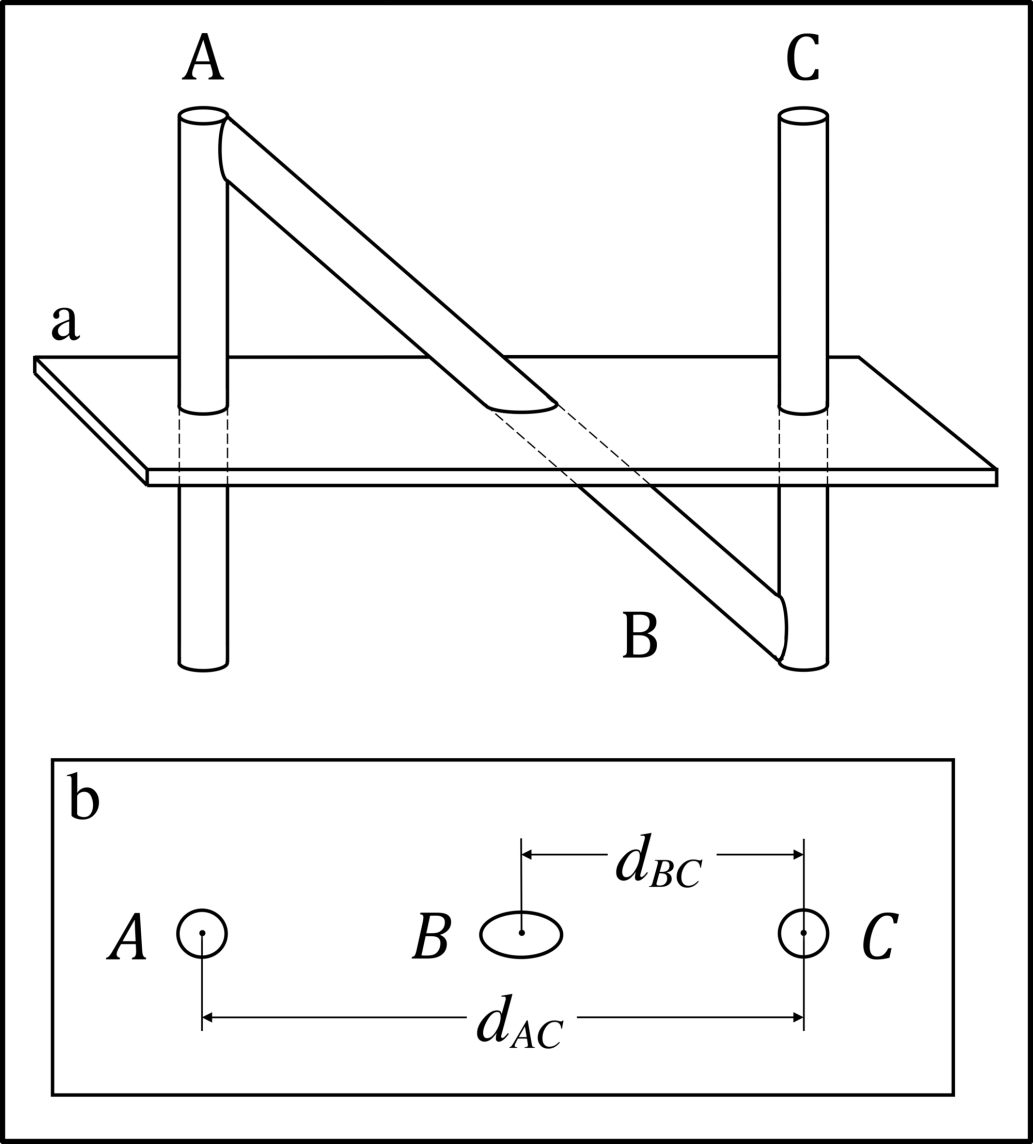
The N-Localizer and its Intersection with a Tomographic Section $\mathbf{(a)}$ Side view of the N-localizer. A tomographic section intersects rods $\mathrm A$, $\mathrm B$, and $\mathrm C$. $\mathbf{(b)}$ Tomographic image. The intersection of the tomographic section with rods $\mathrm A$, $\mathrm B$, and $\mathrm C$ creates fiducial circles $A$ and $C$ and fiducial ellipse $B$ in the tomographic image. The distance $d_{BC}$ between the centers of ellipse $B$ and circle $C$ and the distance $d_{AC}$ between the centers of circles $A$ and $C$ are used to calculate the $z$-coordinate of the point of intersection of the cylindrical axis of rod $\mathrm B$ with the tomographic section [3].

Figure 1 depicts the N-localizer that comprises two vertical rods and one diagonal rod. For the N-localizer, calculation of the $z$-coordinate of the point of intersection of the cylindrical axis of rod $\mathrm B$ with the tomographic section is performed via linear interpolation between the two ends of rod $\mathrm B$ according to the following equation [3]. 

$$z = z_C + \frac{d_{BC}}{d_{AC}}\left ( z_A - z_C \right ) \;\;\;\;\;\; \left(1\right)$$

In this equation, $d_{BC}$ and $d_{AC}$ are distances measured in the $\left(u, v\right)$ coordinate system of the medical image, $z_A$ is the $z$-coordinate of the top of rod $\mathrm A$, and $z_C$ is the $z$-coordinate of the bottom of rod $\mathrm C$. The numeric values for $z_A$ and $z_C$ are established by the manufacturing specifications for the N-localizer. The fraction $d_{BC}/d_{AC}$ is dimensionless, and hence the units of $z$ are the units of $z_A$ and $z_C$ that are specified by the manufacturer. For this reason, calculations for the N-localizer do not require the specification of the pixel size for the medical image [3,4].

**Figure 2:**
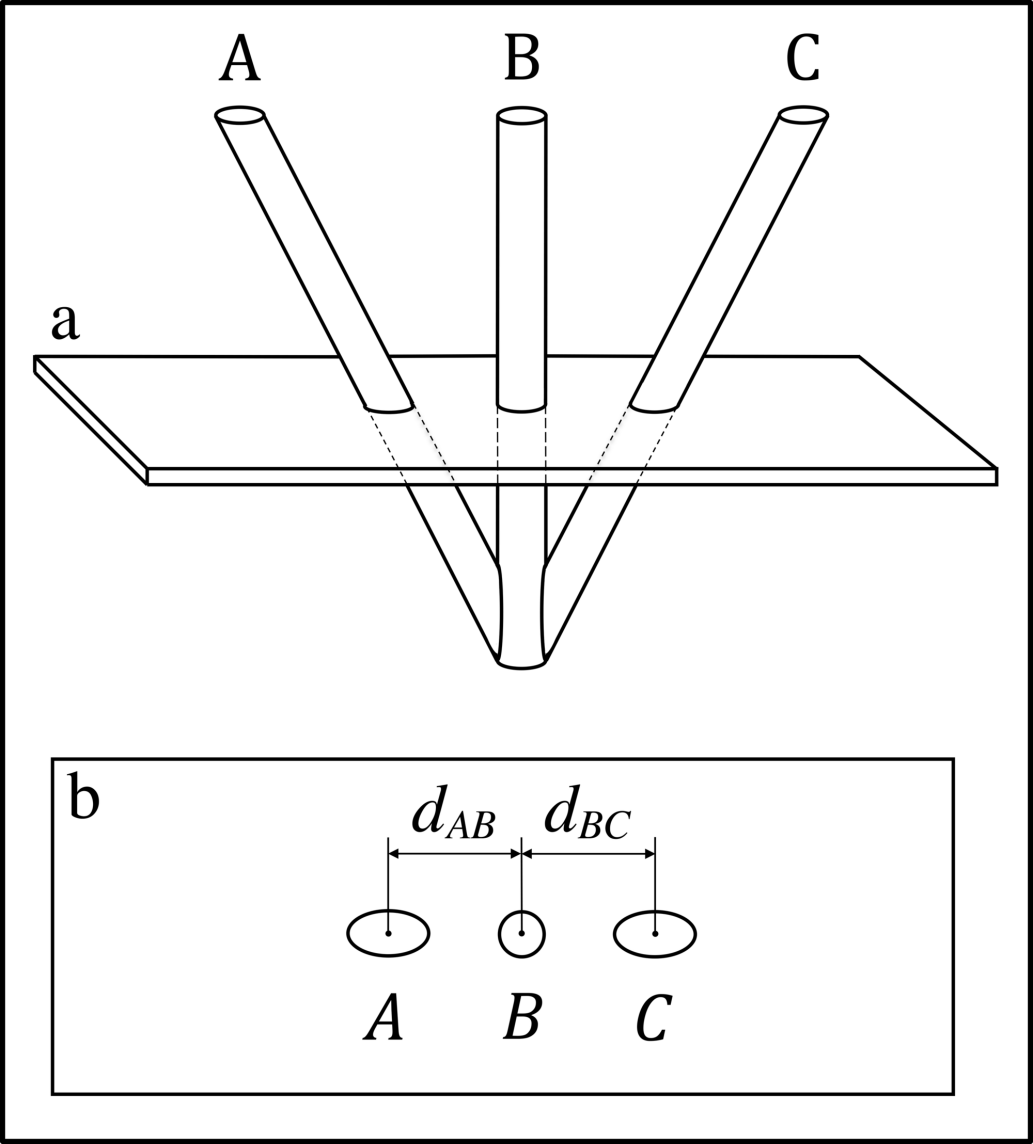
The Sturm-Pastyr Localizer and its Intersection with a Tomographic Section $\mathbf{(a)}$ Side view of the Sturm-Pastyr localizer. A tomographic section intersects rods $\mathrm A$, $\mathrm B$, and $\mathrm C$. $\mathbf{(b)}$ Tomographic image. The intersection of the tomographic section with rods $\mathrm A$, $\mathrm B$, and $\mathrm C$ creates fiducial ellipses $A$ and $C$ and fiducial circle $B$ in the tomographic image. The distance $d_{AB}$ between the centers of ellipse $A$ and circle $B$ and the distance $d_{BC}$ between the centers of circle $B$ and ellipse $C$ are used to calculate the $z$-coordinate of the point of intersection of the cylindrical axis of rod $\mathrm B$ with the tomographic section [6].

Figure 2 depicts the Sturm-Pastyr localizer that comprises two diagonal rods and one vertical rod. For the Sturm-Pastyr localizer, calculation of the $z$-coordinate of the point of intersection of the cylindrical axis of rod $\mathrm B$ with the tomographic section is performed via the following non-linear equation that is derived in the Appendix [5].

$$z = \frac{4d_{AB}d_{BC}}{\sqrt{\left ( d_{BC} + d_{AB} \right )^2 + 4 \left ( d_{BC} - d_{AB} \right )^2}} \;\;\;\; \;\; \left( 2 \right)$$

In this equation, $d_{AB}$ and $d_{BC}$ are distances measured in the $\left(u, v\right)$ coordinate system of the medical image. At the bottom of rod $\mathrm B$, i.e., at the apex of the V-shaped Sturm-Pastyr localizer, $z=0$. When vertical rod $\mathrm B$ is perpendicular to the tomographic section, i.e., when the tomographic section is parallel to the base of the stereotactic frame, Equation (2) reduces to

$$z = d_{AB} + d_{BC} = 2d_{AB} = 2d_{BC} \;\;\;\;\;\; \left( 3 \right)$$

This equation applies because the Sturm-Pastyr localizer is manufactured such that the angle between rods $\mathrm A$ and $\mathrm B$, and the angle between rods $\mathrm B$ and $\mathrm C$, are both $\tan^{-1}\left ( 1/2 \right )$ [6].

Equation (3) requires specification of the pixel size for the medical image to permit conversion of the distances $d_{AB}$ and $d_{BC}$ to millimeters. Equation (2) also requires specification of the pixel size because the units of $z$ calculated by Equation (2) are the units of $d_{AB}$ and $d_{BC}$, as demonstrated by dimensional analysis of Equation (2). This requirement, which does not apply to the N-localizer, renders the Sturm-Pastyr localizer susceptible to error. An erroneous value of $z$ will be calculated via Equations (2, 3) if the pixel size is specified incorrectly via user input, or computed incorrectly from fiducials in the medical image [6], or recorded incorrectly in medical image metadata that require frequent calibration of the imaging system to guarantee correct pixel size.

Figures 1, 2 demonstrate that the tomographic section of a medical image has a finite thickness. It is convenient to ignore this thickness and to approximate a tomographic section as an infinitely thin plane. This "central" plane lies midway between the top and bottom halves of the tomographic section, analogous to the way that a slice of cheese is sandwiched between two slices of bread. In the following presentation, the term "tomographic section" will be used as an abbreviation for the term "central plane of the tomographic section."

Similarly, it is convenient to ignore the diameter of rods $\mathrm A$, $\mathrm B$, and $\mathrm C$ in Figures 1, 2 and to approximate each rod as an infinitely thin cylindrical axis. In the following discussion, the term "rod" will be used as an abbreviation for the term "cylindrical axis of a rod." Hence, in the following presentation, the intersection of a "rod" with a "tomographic section" is equivalent to the intersection of a line with a plane and defines a point.

Monte Carlo algorithm

The accuracies of the N-localizer and Sturm-Pastyr localizer are compared via Monte Carlo simulation that is performed using the following algorithm.

**Figure 3:**
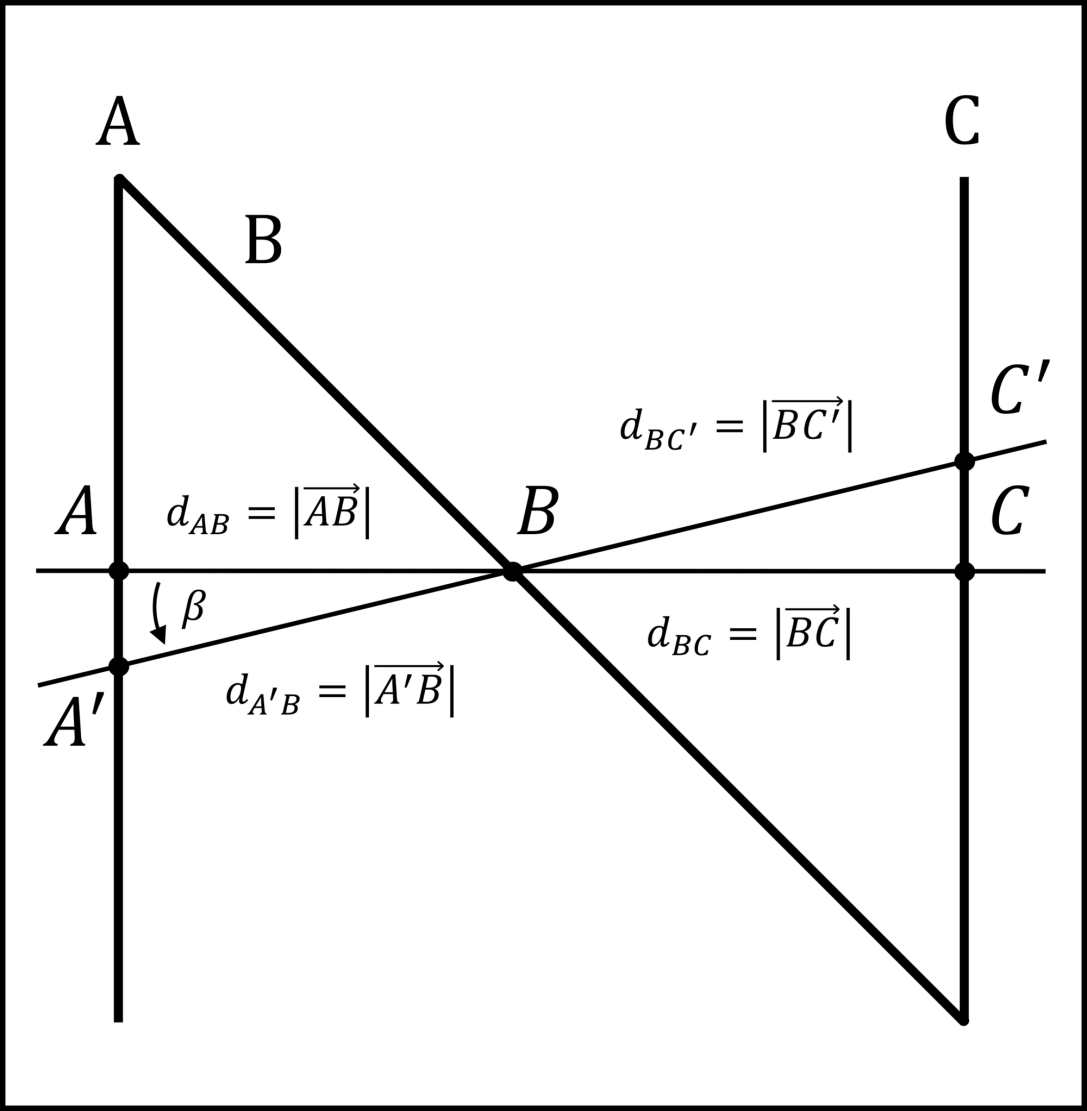
Depiction of the N-Localizer The N-localizer is depicted by rods $\mathrm A$, $\mathrm B$, and $\mathrm C$ that intersect a non-tilted tomographic section at fiducial points $A$, $B$, and $C$. The rods also intersect a tomographic section that is tilted by the angle $\beta$ at fiducial points $A^{'}$, $B$, and $C^{'}$. The distance between points $A$ and $C$ is $d_{AC} = d_{AB} + d_{BC}$. The distance between points $A^{'}$ and $C^{'}$ is $d_{A^{'}C^{'}} = d_{A^{'}B} + d_{BC^{'}}$.

1. A $z$-coordinate is chosen to express the height above the base of the stereotactic frame, i.e., above the base of the localizer.

2. An angle $\beta$ is chosen to express the angle by which the tomographic section is tilted with respect to the localizer such that line $\overline{AC}$ is tilted relative to the base of the stereotactic frame (see Figures 3, 7).

3. The $\left( z, \beta \right)$ pair is used to calculate the $\left( u_A, v_A \right)$, $\left( u_B, v_B \right)$, and $\left( u_C, v_C \right)$ coordinates of the fiducial points $A$, $B$, and $C$, respectively, in millimeters.

4. The $\left( u_A, v_A \right)$, $\left( u_B, v_B \right)$, and $\left( u_C, v_C \right)$ coordinates are perturbed via random numbers [7,8] in the range $\left [ -1 \cdots 1 \right ]$ mm via $n = 2^{25} = 33,554,432$ iterations to create $2^{25}$ sets of perturbed $\left( \hat u^i_A , \hat v^i_A \right)$, $\left( \hat u^i_B, \hat v^i_B \right)$, and $\left( \hat u^i_C, \hat v^i_C \right)$ coordinates, where the superscript $i$ designates the $i$-th perturbed coordinate.

5. Each set of perturbed $\left( \hat u^i_A , \hat v^i_A \right)$, $\left( \hat u^i_B, \hat v^i_B \right)$, and $\left( \hat u^i_C, \hat v^i_C \right)$ coordinates is used to calculate a set of perturbed distances $\hat d^i_{AB}$, $\hat d^i_{BC}$, and $\hat d^i_{AC}$ via the Pythagorean distance equation.

6. Each set of perturbed distances $\hat d^i_{AB}$, $\hat d^i_{BC}$, and $\hat d^i_{AC}$ is used to calculate a perturbed $\hat z_i$-coordinate.

7. The $2^{25}$ perturbed $\hat z_i$-coordinates are used to calculate the root mean square (RMS) error $\sqrt{ \frac{1}{n} \sum_{i}^{n}\left ( z - \hat z_i \right )^2 }$.

8. A new $\left( z, \beta \right)$ pair is chosen and steps 3-7 are repeated.

Monte Carlo simulation for the N-localizer

Step 3 of the Monte Carlo algorithm requires calculation of the $\left( u_A, v_A \right)$, $\left( u_B, v_B \right)$, and $\left( u_C, v_C \right)$ coordinates for a $\left( z, \beta \right)$ pair. To promote clarity, the calculation for a $\left( z, 0 \right)$ pair, for which $\beta = 0$, is discussed first.

Figure 3 depicts an N-localizer wherein rods $\mathrm A$, $\mathrm B$, and $\mathrm C$ intersect both a non-tilted tomographic section, for which $\beta = 0$, and a tilted tomographic section, for which $\beta \neq 0$. For the non-tilted section, calculation of the $\left( u_A, v_A \right)$, $\left( u_B, v_B \right)$, and $\left( u_C, v_C \right)$ coordinates of the respective fiducial points $A$, $B$, and $C$ begins with calculation of the distances $d_{AC}$ and $d_{BC}$. The assumption that vertical rods $\mathrm A$ and $\mathrm C$ are separated by $140$ mm yields $d_{AC} = 140$ mm. The assumption that vertical rods $\mathrm A$ and $\mathrm C$ are $140$ mm high yields $\left( z_A - z_C \right) = 140$ mm. Making the simplification that $z_C = 0$ then yields $d_{BC} = z$ per Equation (1), where $z$ is specified in millimeters.

Given the distances $d_{AC}$ and $d_{BC}$, it is possible to assign values to the $\left( u_A, v_A \right)$, $\left( u_B, v_B \right)$, and $\left( u_C, v_C \right)$ coordinates of the fiducial points $A$, $B$, and $C$. Making the simplification that the fiducial points lie along the $u$-axis, a simple assignment is

$$\left( u_C, v_C \right) = \left( 0, 0 \right) \;\;\;\;\;\;\;\;\;\; \left( u_B, v_B \right) = \left( z, 0 \right) \;\;\;\;\;\;\;\;\;\; \left( u_A, v_A\right) = \left( 140, 0 \right) \;\;\;\;\;\; \left( 4 \right)$$

For the tilted section, calculation of the $\left( u_A, v_A \right)$, $\left( u_B, v_B \right)$, and $\left( u_C, v_C \right)$ coordinates begins with calculation of the distances $d_{A^{'}C^{'}}$ and $d_{BC^{'}}$. Figure 3 reveals that triangles $ABA^{'}$ and $CBC^{'}$ are both right triangles, thus

$$d_{BC^{'}} = \frac{d_{BC}}{\cos \beta} = \frac{z}{\cos \beta} \;\;\;\;\;\;\;\;\;\; d_{A^{'}C^{'}} = \frac{d_{AC}}{\cos \beta} = \frac{140}{\cos \beta} \;\;\;\;\;\; \left( 5 \right)$$

Hence, the $\left( u_A, v_A \right)$, $\left( u_B, v_B \right)$, and $\left( u_C, v_C \right)$ coordinates of the fiducial points $A$, $B$, and $C$ are

$$\left( u_C, v_C \right) = \left( 0, 0 \right) \;\;\;\;\;\;\;\;\;\; \left( u_B, v_B \right) = \left( \frac{z}{\cos \beta}, 0 \right) \;\;\;\;\;\;\;\;\;\; \left( u_A, v_A\right) = \left( \frac{140}{\cos \beta}, 0 \right) \;\;\;\;\;\; \left( 6 \right)$$

Steps 4-7 of the Monte Carlo algorithm then proceed as follows. The $\left( u_A, v_A \right)$, $\left( u_B, v_B \right)$, and $\left( u_C, v_C \right)$ coordinates of the fiducial points $A$, $B$, and $C$ are perturbed $2^{25}$ times by random numbers to obtain $2^{25}$ perturbed $\left( \hat u^i_A , \hat v^i_A \right)$, $\left( \hat u^i_B, \hat v^i_B \right)$, and $\left( \hat u^i_C, \hat v^i_C \right)$ coordinates, from which $2^{25}$ perturbed distances $\hat d^i_{AC}$ and $\hat d^i_{BC}$ are calculated, from which $2^{25}$ perturbed $\hat z_i$-coordinates are calculated via Equation (1) and used to calculate the RMS error $\sqrt{ \frac{1}{n} \sum_{i}^{n}\left ( z - \hat z_i \right )^2 }$. Then a new $\left( z, \beta \right)$ pair is chosen and steps 3-7 of the Monte Carlo algorithm are repeated.

Monte Carlo simulation for the Sturm-Pastyr localizer

For step 3 of the Monte Carlo algorithm applied to the Sturm-Pastyr localizer, calculation of the $\left( u_A, v_A \right)$, $\left( u_B, v_B \right)$, and $\left( u_C, v_C \right)$ coordinates for a $\left( z, \beta \right)$ pair begins with calculation of the distances $d_{AB}$ and $d_{BC}$. For this calculation, Equations (A1, A2) of the Appendix are solved for $d_{AB}$ and $d_{BC}$ to obtain

$$d_{AB} = \frac{z \sin \left ( \upsilon \right )}{\sin \left ( \pi/2+\beta-\upsilon \right )} \;\;\;\;\;\;\;\;\;\; d_{BC} = \frac{z \sin \left ( \upsilon \right )}{\sin \left ( \pi/2-\beta-\upsilon \right )} \;\;\;\;\;\; \left( 7 \right)$$

In these equations, $\upsilon=\tan^{-1}\left ( 1/2 \right )$ for the Sturm-Pastyr localizer [6]. Hence, $d_{AB}$ and $d_{BC}$ are functions of only $z$ and $\beta$.

Given the distances $d_{AB}$ and $d_{BC}$, it is possible to assign values to the $\left( u_A, v_A \right)$, $\left( u_B, v_B \right)$, and $\left( u_C, v_C \right)$ coordinates of the fiducial points $A$, $B$, and $C$. Making the simplification that the fiducial points lie along the $u$-axis, a simple assignment is

$$\left( u_A, v_A \right) = \left( -d_{AB}, 0 \right) \;\;\;\;\;\;\;\;\;\; \left( u_B, v_B \right) = \left( 0, 0 \right) \;\;\;\;\;\;\;\;\;\; \left( u_C, v_C\right) = \left( d_{BC}, 0 \right) \;\;\;\;\;\; \left( 8 \right)$$

Steps 4-7 of the Monte Carlo algorithm then proceed as follows. The $\left( u_A, v_A \right)$, $\left( u_B, v_B \right)$, and $\left( u_C, v_C \right)$ coordinates of the fiducial points $A$, $B$, and $C$ are perturbed $2^{25}$ times by random numbers to obtain $2^{25}$ perturbed $\left( \hat u^i_A , \hat v^i_A \right)$, $\left( \hat u^i_B, \hat v^i_B \right)$, and $\left( \hat u^i_C, \hat v^i_C \right)$ coordinates, from which $2^{25}$ perturbed distances $\hat d^i_{AB}$ and $\hat d^i_{BC}$ are calculated, from which $2^{25}$ perturbed $\hat z_i$-coordinates are calculated via Equation (2) and used to calculate the RMS error $\sqrt{ \frac{1}{n} \sum_{i}^{n}\left ( z - \hat z_i \right )^2 }$. Then a new $\left( z, \beta \right)$ pair is chosen and steps 3-7 of the Monte Carlo algorithm are repeated.

## Discussion

**Figure 4:**
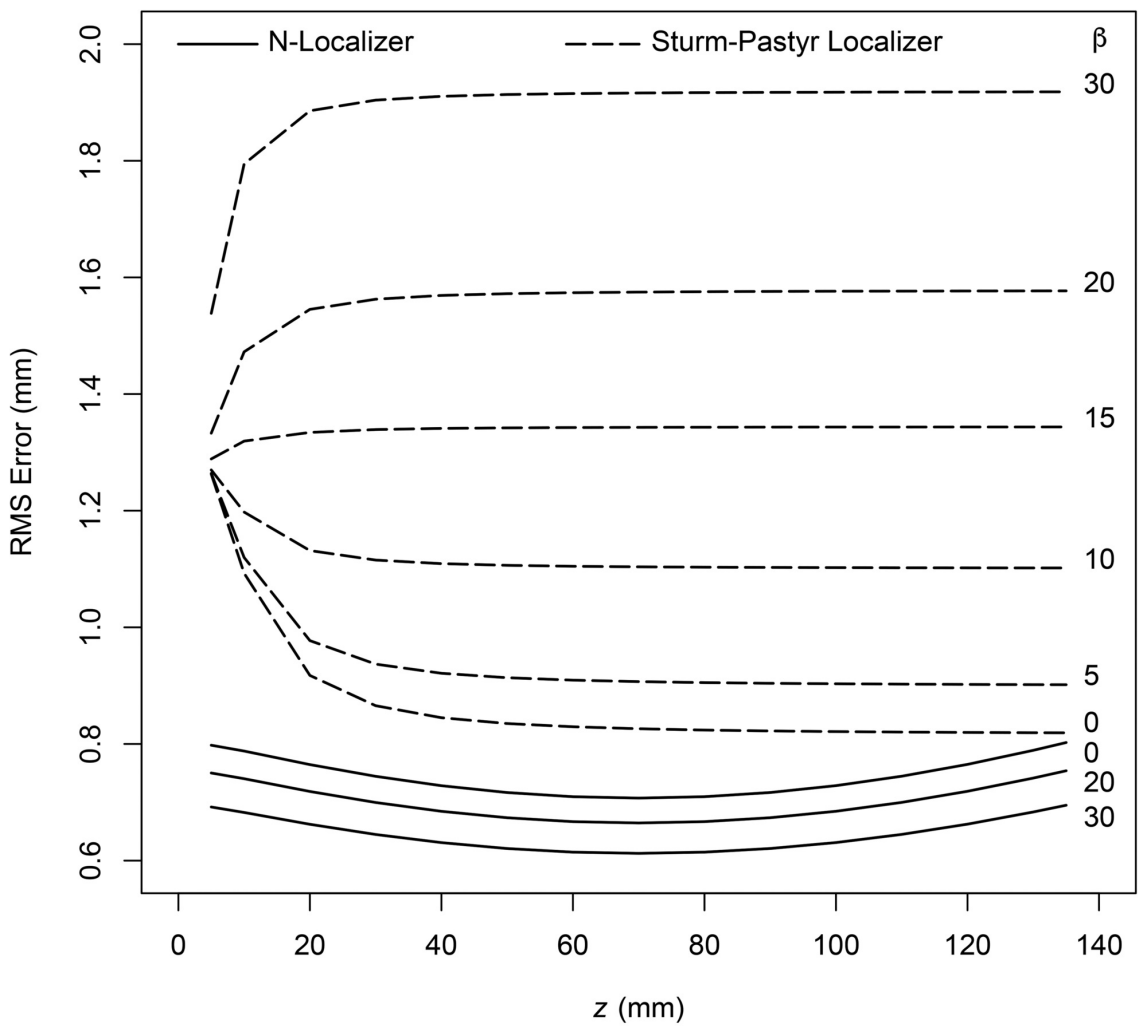
RMS Error in $z$ Plotted vs. $z$ for the N-Localizer and Sturm-Pastyr Localizer The RMS error in $z$ is plotted vs. $z$ for the N-localizer (solid curves) and the Sturm-Pastyr localizer (dashed curves). Each curve is generated using the value of $\beta$ that is specified in degrees to the right of the curve. RMS: root mean square

Figure 4 shows the results of Monte Carlo simulation for the N-localizer and the Sturm-Pastyr localizer. The RMS error in $z$ for the Sturm-Pastyr localizer approaches the smaller RMS error for the N-localizer at only large values of $z$ and tilt angle $\beta=0$. For all other values of $z$ and $\beta$, the Sturm-Pastyr localizer incurs significantly more RMS error than the N-localizer.

The RMS error for the Sturm-Pastyr localizer increases as $z$ decreases and as $\beta$ increases. These trends may be understood by inspecting Equation (7), which shows that $d_{AB}$ is directly proportional to $z$ and inversely proportional to $\sin \left( \pi/2+\beta-\upsilon \right)$; this sine term is maximized when $\beta = \upsilon =\tan^{-1}\left ( 1/2 \right ) = 26.565$ degrees. These trends may also be understood by inspecting Figure 7, which shows that $d_{AB} = \left | \overrightarrow{AB} \right |$ is minimized for a given value of $z = \left | \overrightarrow{OB} \right |$ when line segment $\overline{AB}$ is perpendicular to line segment $\overline{OA}$, i.e., when $\beta = \upsilon$. Thus, an increase in $\beta$ in the range $\left[ 0 \cdots 26.565 \right]$ degrees or a decrease in $z$ decreases $d_{AB}$ and consequently, the random perturbations in the range $\left [ -1 \cdots 1 \right ]$ mm become more significant relative to $d_{AB}$ and thereby increase the RMS error.

**Figure 5:**
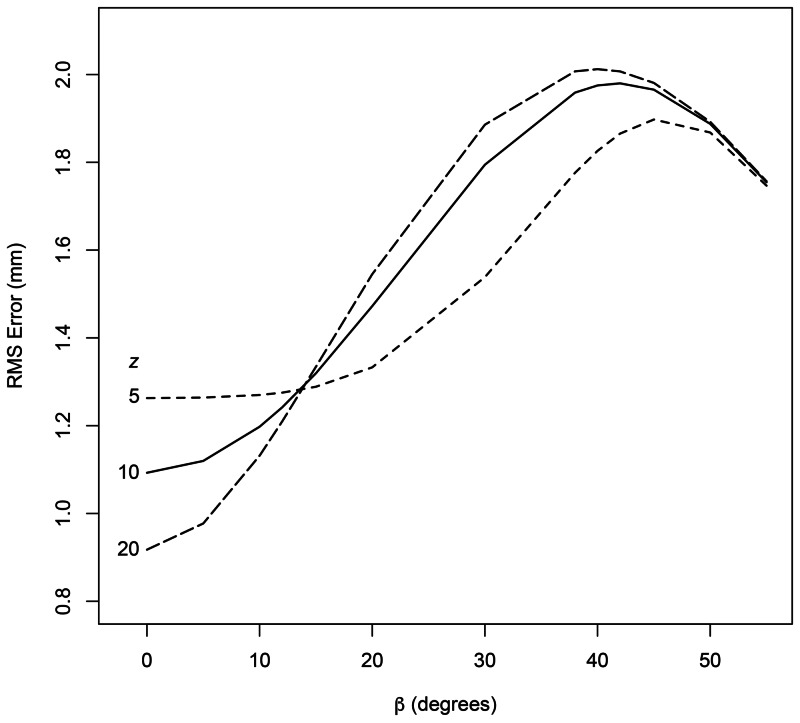
RMS Error in $z$ Plotted vs. $\beta$ for the Sturm-Pastyr Localizer The RMS error in $z$ is plotted versus $\beta$ for the Sturm-Pastyr localizer. Each curve is generated using the value of $z$ that is specified in millimeters to the left of the curve. The curves for $z\gt20$ mm are similar to the curve for $z=20$ mm and are omitted. RMS: root mean square

Equation (7) also shows that $d_{BC}$ is inversely proportional to $\sin \left( \pi/2-\beta-\upsilon \right)$ and hence increases monotonically as $\beta$ increases in the range $\left [ 0 \cdots 63.435 \right ]$ degrees, where $63.435 = 90 - \upsilon$. And Equation (2) shows that $z$ depends on $d_{BC}$ and $d_{AB}$ in a non-linear manner. Figure 5 demonstrates the effect of this non-linearity on the RMS error in $z$ for the Sturm-Pastyr localizer and reveals that the maximum RMS error occurs near $\beta = 40$ degrees.

The RMS error for the N-localizer decreases as $\beta$ increases. This trend may be understood by inspecting Equation (6), which shows that the unperturbed $\left( u_A, v_A \right)$, $\left( u_B, v_B \right)$, and $\left( u_C, v_C \right)$ coordinates are inversely proportional to $\cos \left( \beta \right)$. Hence, as $\beta$ increases in the range $\left [ 0 \cdots 90 \right ]$ degrees, the unperturbed coordinates increase as well and in consequence, the random perturbations in the range $\left [ -1 \cdots 1 \right ]$ mm become less significant relative to the magnitudes of the unperturbed coordinates and thereby decrease the RMS error.

Random perturbations in the range $\left [ -1 \cdots 1 \right ]$ mm are used for the Monte Carlo algorithm due to the following considerations. A typical field of view (FOV) for a medical image that is used for planning stereotactic surgery lies in the range $\left [ 250 \cdots 360 \right ]$ mm and comprises 512x512 pixels. Hence, the pixel size for such an image is in the range $\left [ 0.5 \cdots 0.7 \right ]$ mm. A conservative estimate that the center of each fiducial circle or ellipse is displaced at most two pixels by random noise yields the perturbation range $\left [ -1 \cdots 1 \right ]$ mm.

**Figure 6:**
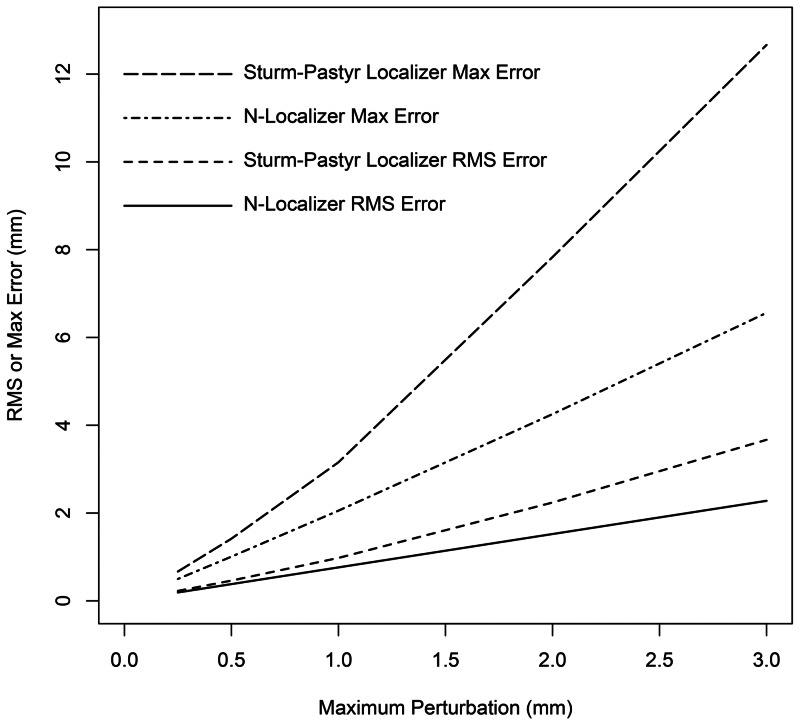
RMS and Maximum Errors vs. Maximum Perturbation for the N-Localizer and Sturm-Pastyr Localizer at $z = 20$ mm and $\beta = 5$ Degrees The RMS and maximum errors are plotted vs. the maximum perturbation for the N-localizer (solid and dot-dashed curves) and the Sturm-Pastyr localizer (dashed and long-dashed curves) at $z = 20$ mm and $\beta = 5$ degrees. RMS: root mean square

**Figure 7:**
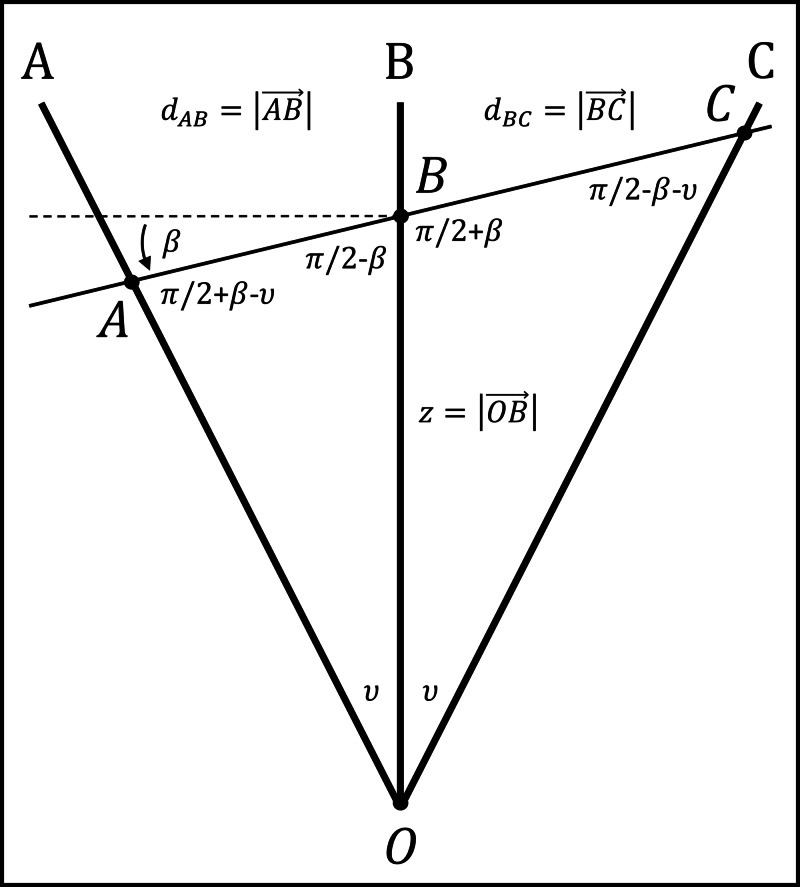
Depiction of the Sturm-Pastyr Localizer The Sturm-Pastyr localizer is depicted by rods $\mathrm A$, $\mathrm B$, and $\mathrm C$ that intersect the tomographic section at fiducial points $A$, $B$, and $C$. The tomographic section is tilted by $\beta$ degrees. The distance between points $O$ and $B$ is $z$. The distance between points $A$ and $B$ is $d_{AB}$. The distance between points $B$ and $C$ is $d_{BC}$. Because angle $\upsilon$ is a constant for the Sturm-Pastyr localizer, i.e., $\upsilon = \tan^{-1}\left ( 1/2 \right )=26.565$ degrees [6], angles $OAB$, $ABO$, $OBC$, and $BCO$ are functions of only angle $\beta$, e.g., angle $BCO=\pi/2-\beta-\upsilon$.

The effect of various perturbation ranges on the errors incurred by the N-localizer and Sturm-Pastyr localizer is shown in Figure 6. This figure plots the RMS and maximum errors for both localizers at $z=20$ mm and $\beta=5$ degrees vs. the maximum perturbation for the following continuous ranges of white noise: $\left [ -0.25 \cdots 0.25 \right ]$, $\left [ -0.5 \cdots 0.5 \right ]$, $\left [ -1 \cdots 1 \right ]$, $\left [ -2 \cdots 2 \right ]$, and $\left [ -3 \cdots 3 \right ]$ mm. The RMS and maximum errors for the N-localizer scale linearly with the maximum perturbation: the slope and correlation coefficient of a linear least-squares fit to the RMS-error data are 0.76 and 0.999991, respectively; the slope and correlation coefficient of a linear least-squares fit to the​​​​​​​ maximum-error data are 2.21 and 0.9998, respectively. As can be appreciated from Figure 6, the RMS and maximum errors for the Sturm-Pastyr localizer scale slightly super-linearly, as demonstrated by the slight upward concavity of the Sturm-Pastyr curves. The combination $z=20$ mm and $\beta=5$ degrees pertains to a medical image that is obtained near the base of the stereotactic frame and almost parallel to the base of the frame. Such an image would be acquired for functional neurosurgery of the basal ganglia or for the insertion of deep brain stimulation implants.

## Conclusions

The Sturm-Pastyr localizer was originally intended for use with a medical image that is parallel to the base of the stereotactic frame, as depicted in Figure 2, wherein vertical rod $\mathrm B$ is perpendicular to the tomographic section. Obtaining such a parallel image is difficult because it requires precise alignment of the patient. The equations presented in the Appendix extend this localizer for use with a medical image that is not parallel to the base of the stereotactic frame. But these equations cannot surmount the V-shape of the Sturm-Pastyr localizer that hampers its accuracy for a non-parallel image. And, even for a parallel image, the accuracy of this localizer degrades substantially near the apex of the V, i.e., near the base of the stereotactic frame. This decreased accuracy may hinder the effectiveness of the Sturm-Pastyr localizer for targets deep in the brain, e.g., for functional neurosurgery of the basal ganglia or for insertion of deep brain stimulation implants.

In contrast to the Sturm-Pastyr localizer, the N-localizer is intended for use with a medical image that is not perforce parallel to the base of the stereotactic frame. Hence, there is no requirement to precisely align the patient to obtain a parallel image. In fact, the accuracy of the N-localizer increases for a non-parallel image. And for either parallel or non-parallel images, the N-localizer is more accurate than the Sturm-Pastyr localizer. An additional advantage of the N-localizer compared to the Sturm-Pastyr localizer is that the N-localizer does not require specification of the pixel size for a medical image.

## Appendices

The Sturm-Pastyr localizer is designed to provide the $z$-coordinate when vertical rod $\mathrm B$ of the localizer is perpendicular to the tomographic section, i.e., when the tomographic section is parallel to the base of the stereotactic frame. This idealized case is depicted in Figure 2 but not in Figure 7. In the idealized case, $d_{AB}=d_{BC}$ and $z=d_{AB}+d_{BC}$ because angles $AOB$ and $BOC$ shown in Figure 7 are both $\upsilon=\tan^{-1}\left ( 1/2 \right )=26.565$ degrees [6]. However, achieving the idealized case is impractical due to the difficulty of precisely aligning the patient such that the tomographic section is perpendicular to vertical rod $\mathrm B$. Moreover, image noise perturbs the distances $d_{AB}$ and $d_{BC}$ such that $d_{AB}\neq d_{BC}$ even if the patient is precisely aligned. For these reasons, Dai et al. have derived equations that permit calculation of $z$ from $d_{AB}$ and $d_{BC}$ when the tomographic section is not perpendicular to vertical rod $\mathrm B$ [6].

Understanding the derivation of Dai, et al. requires familiarity with only trigonometry and algebra. However, because Dai, et al. omitted several intermediate steps from their derivation, it is unnecessarily obscure. The intermediate steps are provided below and in addition, the result reported by Dai, et al. is extended to yield an expression that contains no trigonometric functions.

The derivation of Dai, et al. produces an equation for the angle $\beta$ in terms of the distances $d_{AB}$ and $d_{BC}$ as follows.

Application of the law of sines to triangle $OAB$ of Figure 7 yields 

$$\frac{z}{\sin \left ( \pi/2+\beta-\upsilon \right )} = \frac{d_{AB}}{\sin \left ( \upsilon \right )} \;\;\;\;\;\; \left( A1 \right)$$

In this equation, $z=\left | \overrightarrow{OB} \right |$ and $d_{AB}=\left | \overrightarrow{AB} \right |$.

Similarly, application of the law of sines to triangle $OBC$ yields 

$$\frac{z}{\sin \left ( \pi/2-\beta-\upsilon \right )} = \frac{d_{BC}}{\sin \left ( \upsilon \right )} \;\;\;\;\;\; \left( A2 \right)$$

In this equation, $z=\left | \overrightarrow{OB} \right |$ and $d_{BC}=\left | \overrightarrow{BC} \right |$.

Dividing Equation (A1) by Equation (A2) eliminates $z$ and $\sin \left ( \upsilon \right )$ to yield

$$\frac{\sin \left ( \pi/2-\beta-\upsilon \right )}{\sin \left ( \pi/2+\beta-\upsilon \right )} = \frac{d_{AB}}{d_{BC}} \;\;\;\;\;\;\ \left( A3 \right)$$

Applying the rule for the sine of the sum of two angles followed by the rule for the cosine of the sum of two angles produces 

$$\frac{\cos \left ( \beta+\upsilon \right )}{\cos \left ( \beta-\upsilon \right )} = \frac{\cos \left ( \beta \right ) \cos \left ( \upsilon \right ) - \sin \left ( \beta \right ) \sin \left ( \upsilon \right )}{\cos \left ( \beta \right ) \cos \left ( \upsilon \right ) + \sin \left ( \beta \right ) \sin \left ( \upsilon \right )} = \frac{d_{AB}}{d_{BC}} \;\;\;\;\;\; \left( A4 \right)$$

Cross multiplication by the denominators and then factoring in $\sin \left( \beta \right) \sin \left( \upsilon \right)$ and $\cos \left( \beta \right) \cos \left( \upsilon \right)$ yields

$$\frac{d_{BC} - d_{AB}}{d_{BC} + d_{AB}} = \frac{ \sin \left( \beta \right) \sin \left( \upsilon \right) }{ \cos \left( \beta \right) \cos \left( \upsilon \right) } = \tan \left ( \beta \right ) \tan \left ( \upsilon \right ) \;\;\;\;\;\; \left ( A5 \right )$$

Substituting $\tan \left ( \upsilon \right ) = 1/2$ into Equation (A5) yields

$$\tan \left ( \beta \right ) = \frac{2 \left ( d_{BC} - d_{AB} \right )}{d_{BC} + d_{AB}} \;\;\;\;\;\; \left ( A6 \right )$$

Solving Equation (A6) for $\beta$ completes the derivation of Dai, et al [6].

$$\beta = \tan^{-1} \left [ \frac{2 \left ( d_{BC} - d_{AB} \right )}{d_{BC} + d_{AB}} \right ] \;\;\;\;\;\; \left ( A7 \right )$$

Solving Equation (A1) for $z$ yields

$$z = \frac{d_{AB} \sin \left ( \pi/2+\beta-\upsilon \right )}{\sin \left ( \upsilon \right )}\;\;\;\;\;\; \left ( A8 \right )$$

Computing $\beta$ via Equation (A7) and substituting $\beta$ into Equation (A8) calculates $z$ in terms of $d_{AB}$ and $d_{BC}$ when the tomographic section is not perpendicular to vertical rod $\mathrm B$.

An expression for $z$ in terms $d_{AB}$ and $d_{BC}$ that does not involve trigonometric functions is obtained by first constructing expressions for $\sin \left ( \beta \right )$ and $\cos \left ( \beta \right )$ via inspection of Equation (A6)

$$\sin\left ( \beta \right ) = \frac{2 \left ( d_{BC} - d_{AB} \right )}{\sqrt{\left ( d_{BC} + d_{AB} \right )^2 + 4 \left ( d_{BC} - d_{AB} \right )^2}} \;\;\;\;\;\;\;\;\;\; \cos \left ( \beta \right ) = \frac{d_{BC} + d_{AB} }{\sqrt{\left ( d_{BC} + d_{AB} \right )^2 + 4 \left ( d_{BC} - d_{AB} \right )^2}} \;\;\;\;\;\; \left ( A9 \right )$$

Applying the rules for the sine and cosine of the sum of two angles to Equation (A8) yields

$$z = \frac{d_{AB} \left [ \cos \left ( \beta \right ) \cos \left ( \upsilon \right ) + \sin \left ( \beta \right ) \sin \left ( \upsilon \right ) \right ]}{\sin \left ( \upsilon \right )} = d_{AB} \left [ \frac{ \cos \left ( \beta \right )}{ \tan \left ( \upsilon \right )} + \sin \left ( \beta \right )\right ] \;\;\;\;\;\; \left ( A10 \right )$$

Substituting $\tan \left ( \upsilon \right ) = 1/2$ into Equation (A10) yields

$$z= d_{AB} \left [ 2 \cos \left ( \beta \right ) + \sin \left ( \beta \right )\right ] \;\;\;\;\;\; \left ( A11 \right )$$

Substituting Equation (A9) into Equation (A11) yields a non-linear expression for $z$ in terms $d_{AB}$ and $d_{BC}$ that does not involve trigonometric functions [5]

$$z = \frac{4d_{AB}d_{BC}}{\sqrt{\left ( d_{BC} + d_{AB} \right )^2 + 4 \left ( d_{BC} - d_{AB} \right )^2}} \;\;\;\; \;\; \left( A12 \right)$$

When $d_{AB} = d_{BC}$, Equation (A12) reduces to Equation (3).
